# Dopamine Beta Hydroxylase Genotype Identifies Individuals Less Susceptible to Bias in Computer-Assisted Decision Making

**DOI:** 10.1371/journal.pone.0039675

**Published:** 2012-06-27

**Authors:** Raja Parasuraman, Ewart de Visser, Ming-Kuan Lin, Pamela M. Greenwood

**Affiliations:** 1 Center of Excellence in Neuroergonomics, Technology, and Cognition (CENTEC) and Department of Psychology, George Mason University, Fairfax, Virginia, United States of America; 2 Department of Molecular Neuroscience, George Mason University, Fairfax, Virginia, United States of America; University of Queensland, Australia

## Abstract

Computerized aiding systems can assist human decision makers in complex tasks but can impair performance when they provide incorrect advice that humans erroneously follow, a phenomenon known as “automation bias.” The extent to which people exhibit automation bias varies significantly and may reflect inter-individual variation in the capacity of working memory and the efficiency of executive function, both of which are highly heritable and under dopaminergic and noradrenergic control in prefrontal cortex. The dopamine beta hydroxylase (DBH) gene is thought to regulate the differential availability of dopamine and norepinephrine in prefrontal cortex. We therefore examined decision-making performance under imperfect computer aiding in 100 participants performing a simulated command and control task. Based on two single nucleotide polymorphism (SNPs) of the DBH gene, −1041 C/T (rs1611115) and 444 G/A (rs1108580), participants were divided into groups of low and high DBH enzyme activity, where low enzyme activity is associated with greater dopamine relative to norepinephrine levels in cortex. Compared to those in the high DBH enzyme activity group, individuals in the low DBH enzyme activity group were more accurate and speedier in their decisions when incorrect advice was given and verified automation recommendations more frequently. These results indicate that a gene that regulates relative prefrontal cortex dopamine availability, DBH, can identify those individuals who are less susceptible to bias in using computerized decision-aiding systems.

## Introduction

Computers are increasingly being used as “intelligent aids” to assist decision makers in their work. Examples include: a radiologist using a computer detection system to decide whether a mammogram is normal or contains a cancerous tumor [1]; an airline pilot employing an electronic flight planner to decide which route to fly [2]; or an administrator using software to decide whether an individual should receive unemployment or healthcare benefits [3]. The use of such automated tools frequently helps speed up decision time, thereby boosting efficiency and throughput. Yet automation can sometimes provide faulty advice to the user. If the human uncritically accepts the computer's decision on such an occurrence–a tendency called “automation bias” [4] that has been likened to a decision heuristic [5], [6]–the consequences can be severe for those affected by the erroneous decision [7]. In extreme cases, the outcome could be catastrophic, as in the instance of military personnel wrongly following a decision aid's recommendation to direct missiles to a target, resulting in fratricide or civilian casualties [8].

Automation bias reflects a tendency for people to rely on and accept computerized decision advice without checking information sources that would confirm or disconfirm the automated advisory [4], [9], [10]. The propensity reflects a user's *perceived* reliability of an automated system and not necessarily its actual capability. Because automated systems are dependent on inputs that can be noisy (e.g., sensor data), they may give unreliable advice to the user even if their algorithms are 100% capable [7]. Automation bias is widespread and not diminished with domain expertise [11] or by exhortations to users to be accountable [12]. Yet there are significant differences between people in the extent to which they exhibit automation bias. Some are very susceptible, others not so much. What is the source of such differences? One possibility is inter-individual variation in cognitive components underlying speeded decision-making, particularly working memory and executive function.

Twin studies have shown that both working memory [13] and executive function [14] are strongly heritable, suggesting that normal variation in genes may contribute to individual differences in these cognitive functions. Molecular genetic methods can be used to examine such inter-individual variability [15]–[18]. The prefrontal cortex plays a critical role in working memory and executive function and in the contribution of those functions to effective decision-making [19]–[21]. Neural activity in this brain region is modulated by two important neurotransmitters, dopamine (DA) and norepinephrine (NE). DA and NE activity in the prefrontal cortex have been linked to simple match-to-sample decisions in working memory tasks [22]–[24]. Pharmacological studies in monkeys have also linked DA and NE activity in a dose-dependent manner to working memory performance [23], [25], [26]. We therefore hypothesized that genes that code for the relative availability of DA and NE would be associated with individual differences in complex decision making under (imperfect) computer aiding.

**Figure 1:**
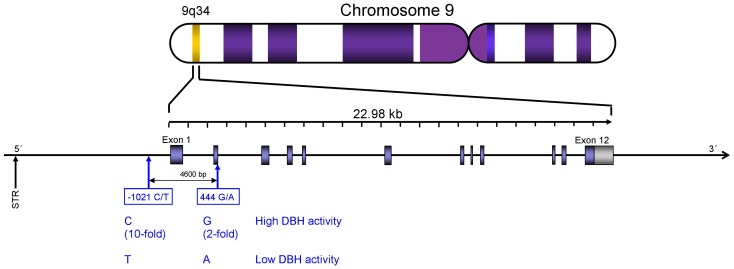
A representation of the DBH gene, which is found on chromosome 9. The locations of the −1021 C/T (rs1611115) and 444 G/A (rs1108580) SNPs, and their associations with DBH enzyme activity are also shown.

The dopamine beta hydroxylase (DBH) gene regulates the differential availability of DA and NE in cortex [27], [28]. Two of the more important variants or single nucleotide polymorphisms (SNPs) in the DBH gene are the −1021 C/T SNP (rs1611115), which is found 1021 bp upstream in the promoter region of the DBH gene, and the 444 G/A SNP (rs1108580), which occurs 444 bp downstream in exon 2 of the gene (see Figure 1). We have previously shown that the 444 G/A SNP is associated with individual differences in retention accuracy in a spatial working memory (match-to-sample) task but not with performance on a spatial attention task [29], [30]. In the present study we investigated whether the association reported in these previous studies between DBH and very simple decision making–deciding whether a probe dot presented at a particular location matches one of up to three locations held briefly in mind–also holds for a more complex, dynamic task more representative of decision making in work settings with computerized decision aids.

The DBH gene is found on chromosome 9 and is about 23,000 base pairs (bp) long (see Figure 1). Post-synaptic DA and NE levels are strongly associated with DBH enzyme activity since DBH is expressed specifically in NE-containing neurons and is the only catecholamine-synthetic enzyme located within synaptic vehicles [27], [28]. The −1021 C/T SNP is associated with a ∼10 fold change in plasma DBH enzyme activity and the 444 G/A SNP with a ∼3 fold change [28]. Plasma and cerebrospinal fluid (CSF) DBH levels are highly correlated at about 0.7 [31] and are correlated with plasma levels of immunoreactive DBH protein [32]. A twin study found that the heritability of plasma DBH was 0.98 while that of CSF DBH was 0.83 [33]. Furthermore the rs1108580 SNP is significantly associated with both plasma and CSF DBH enzyme levels [27]. High DBH enzyme activity is thought to lead to greater conversion of DA to NE in the synapse, and therefore to lower post-synaptic DA compared to NE levels; conversely low enzyme activity is associated with greater DA compared to NE levels [28]. Given a link between increased DA levels and decision-making performance [19]–[21], we therefore expected low DBH enzyme activity to be associated with superior performance when computer assistance was not perfectly reliable. A reviewer suggested that our hypothesis of a link between DBH and dopamine activity ignores the possible role of dopaminergic neurons. Evidence consistent with the hypothesized link comes from a study of patients with Parkinson's Disease (PD), the quintessential pathological condition of dopamine depletion: a strong association was reported between DBH and PD for the same SNP we examined, rs1611115, for the C allele of this SNP, consistent with an association with low dopaminergic function [34]. Furthermore, Weinshenker and colleagues [35] showed that DBH knockout mice were hypersensitive to amphetamine due to changes in the sensitivity of dopamine signaling, given that they were relatively insensitive to a D1 agonist and hypersensitive to a D2 agonist. The authors concluded that DBH affects dopamine signaling pathways. Based on this human and animal evidence that the DBH gene does affect dopamine signaling, we hypothesized a link between DBH and dopamine activity.

The thymine (T) allele of the −1021 C/T SNP and the adenine (A) allele of the 444 G/A SNP are associated with lower DBH enzyme activity. Therefore, assuming additive effects of the two SNPs, which are in linkage disequilibrium, we predicted that individuals with two copies of the T allele of the −1021 C/T SNP (TT) and two copies of the A allele of the 444 G/A SNP (AA) would show the lowest DBH enzyme activity and the best decision-making performance compared to individuals with the CC and GG genotypes on these SNPs.

We used a simulated military command and control task previously used in a study examining the effects of imperfect automation on complex decision-making [36]. This task involves not only spatial processing, as in the simpler spatial working memory task used in the DBH association study [29], but also requires participants to make judgments about the relative positions of “friendly” and “enemy” units under time pressure. The task also includes an automated decision aid that participants can choose to rely on or not. Imperfect decision aiding was manipulated by having the automated advisories be always correct (100%), or in a separate block of trials, 80% correct. Participants had the option to verify the automation recommendation before making their decision choice by clicking on an “Information” button. Participants also performed the task manually, without decision support. We predicted that when given imperfect decision support (80% automation reliability), decision accuracy would be lower on unreliable trials than on reliable trials–the automation bias effect–but that the low DBH enzyme activity group would be more accurate and faster in decision making on unreliable trials than the high enzyme activity group. Given that Bahner and colleagues [9] found that individuals not exhibiting automation bias verified more information parameters than those who did, we also expected the low DBH enzyme activity group to use the verification option more frequently and express lower trust in the decision aid when it gave wrong advice.

## Materials and Methods

### Ethics Statement

All human participants provided informed consent to take part in the study, which was approved by the George Mason University Institutional Review Board.

### Participants

One hundred adults were selected from a sample of 795 individuals who were genotyped for the −1021 C/T (rs1611115) and 444 G/A (rs1108580) SNPs of the DBH gene. Each SNP was found to be in Hardy-Weinberg Equilibrium in the larger sample (rs1611115: *p* = .13; rs1108580: *p* = .28). The 100 selected individuals were chosen with genotypes so as to form two groups, a low DBH enzyme activity group and a high DBH enzyme activity group. Increasing T dose of the −1021 C/T SNP is associated with a decrease in plasma DBH enzyme activity [28]. Therefore, TT homozygotes have the lowest level of DBH enzyme, followed by individuals with the CT and CC genotypes. Also, given that increasing A dose of the 444 G/A SNP is associated with a decrease in DBH enzyme activity, AA homozygotes have lower enzyme activity levels than individuals with AG and GG genotypes. Accordingly, we selected TT homozygotes on the −1021 C/T SNP who were also AA homozygotes on the 444 G/A SNP to form a low DBH enzyme activity group (TT+AA combination). Using similar reasoning, we formed a high DBH enzyme activity group by selecting participants who were CC homozygotes on the −1021 C/T SNP and also GG homozygotes on the 444 G/A SNP (CC+GG combination). This selective genotype approach is similar to that used in a previous study of DBH [37]. The low DBH enzyme activity group included 50 individuals (24 males, 26 females) aged 18–27 years (mean = 20.7). The high DBH enzyme activity group included 50 individuals (23 males, 27 females) aged 18–28 years (mean = 20.7).

### Genotyping

After informed consent, genomic material was obtained via buccal swabs and DNA was prepared with the BuccalAmp™ DNA Extraction Kit (Epicenter Biotechnologies). Each individual was genotyped for the rs1611115 (−1021C/T) and rs1108580 (444G/A) SNPs of the DBH gene using a combination of nested polymer chain reaction (PCR) and DNA melting curve analysis with T_m_-shift primers [38], [39]. The amplicon of the first round PCR was used as a template in a second round real-time PCR (Bio-rad MyiQ thermal cycler) for automated melting curve analysis. In real-time PCR, two allele-specific forward primers, one with a GC-rich tail at the 5′ end, in addition to a common reverse primer, were designed for each SNP, so that the 3′ end of the allele-specific primers coincided with the SNP position [38]. PCR reaction conditions were optimized for each primer pair. Participant genotypes were further confirmed by repeated scoring and/or DNA sequencing.

### Automated Command and Control Task

**Figure 2:**
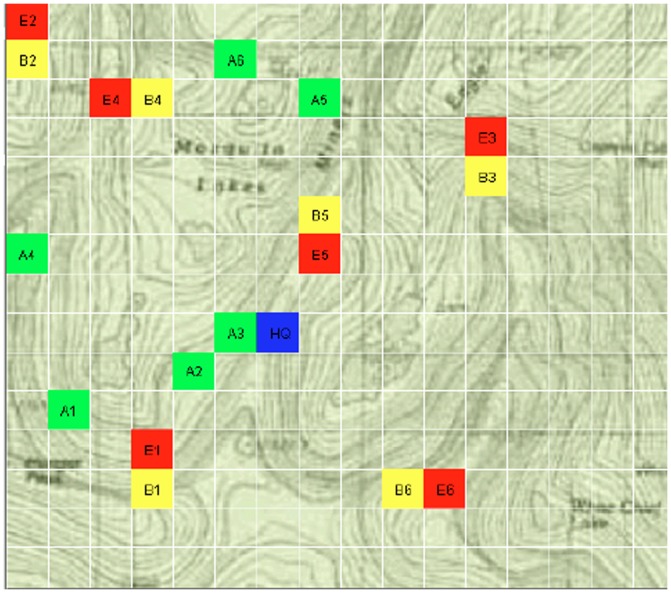
Screen shot of terrain map in the command and control decision–making task showing artillery (green), battalion (yellow), enemy (red), and HQ (blue) units.

The simulated command and control task was presented on a 17 in (43 cm) color monitor, with a mouse used as an input device. The task display had three separate parts: a terrain map, a response window, and an automation recommendation and information window. The right portion of the screen was dedicated to a two-dimensional terrain view displaying three red enemy units (labeled E1, E2, and E3), three yellow friendly battalion units (B1, B2, and B3), six green friendly artillery units (A1, A2, A3, A4, A5, and A6), and one blue friendly headquarter unit (HQ) (See Figure 2). A smaller window to the left of the terrain window contained a response area where the user made enemy-friendly engagement selection. Participants were required to identify the most dangerous enemy target and to select a corresponding friendly unit to engage in combat with the target. The criteria for enemy unit engagement selection (derived by consulting with military subject matter experts) was based not only on the closest distance between it and friendly units but also the relative distance to the HQ unit, with a red unit that was closer to the HQ than another red unit classified as more dangerous and requiring engagement. Specifically, the following criteria had to be met: 1. Only artillery units could engage enemy units in combat. 2. Enemy units had to be within 20 km (in east, west, north, or south directions) of the friendly unit to be considered as an appropriate choice for engagement. 3. The friendly unit closest in distance to an enemy unit was to be given the highest priority for combat engagement. 4. If two friendly units were equally distant from an enemy unit, or if a friendly unit could engage in combat with two enemy units that were both an equal distance away from the friendly unit then it was important to select the unit closest to headquarters.

The automation state part of the display provided the participant with a recommendation of the best enemy-friendly engagement selection (e.g., “E2-A3”), with the automation algorithm taking into account distances between enemy targets, friendly units, and headquarters, as described above. Participants could choose to follow the automation recommendation or make their own decision regarding enemy-friendly unit engagement. If they wished, participants could verify the automation recommendation before making their decision choice by clicking on an “Information” button, in which case the distances between the recommended enemy, friendly, and HQ units were displayed. Participants were required to make a decision within 10 s. After they clicked the response button, or if 10 s had elapsed, the trial ended and the terrain map was replaced with a new set of locations of enemy, friendly, and HQ units.

### Procedure

Participants were first familiarized with the command and control decision-making task and given examples of correct enemy-target engagement selection choices. During this training phase, the display did not show the automation support window. Participants were then given 20 trials of practice on the task. If they did not achieve a criterion performance level of 70% correct decision choices, they were given another block of 20 practice trials. Following the practice period, participants performed the task for 50 trials without automated support. This was the “Manual” condition. After this they were shown the task with the automation support window present and given examples of automation recommendations and the “verification” procedure. Participants then performed the task for 50 trials with automation support. The automation recommendations provided during this block of trials were always correct. This was the “Automation-100%” condition. Participants then completed 200 trials in two blocks of 100 trials each (with a rest break in between blocks) in which the automation recommendations were correct 80% of the time. This was the “Automation-80%” condition. Thus, on 160 trials (80%) the automation recommendation was the correct one, whereas on 40 trials (20%) an incorrect recommendation was given. Prior to the automation blocks, participants were told that the automation recommendation was highly but not perfectly reliable. (No other information on automation reliability was given.) Participants rated their trust in the automation recommendation on a scale of 1–10 after the Automation-100% and Automation-80% blocks of trials.

## Results

### Data analyses

Dependent variables on the command and control task included the accuracy and speed of enemy-friendly engagement selections. Accuracy was calculated by the percentage of trials in which the participant correctly selected the most dangerous enemy target and a corresponding friendly unit to engage. In addition, the proportion of trials on which participations clicked on the information verification window was also computed in the automation conditions. Mean decision accuracy and decision time were computed for each DBH genotype group for the manual and the two automation conditions (100% and 80% reliability). These were then analyzed in 2 (genotype group, low or high DBH enzyme activity)×3 (Manual, Automation-100%, Automation-80%) analyses of variance (ANOVAs). For the imperfect (80%) automation condition, decision-making performance was first computed for all trials, reliable and unreliable. In subsequent analyses, decision-making performance measures were computed separately for reliable (80%) and unreliable (20%) trials. These were then subjected to 2 (genotype group)×2 (reliable, unreliable) ANOVAs. The verification rate measure was analyzed in a 2 (genotype group)×3 (Automation-100%, Automation-80% reliable, unreliable) ANOVA. Finally, subjective trust was analyzed in a 2 (genotype group)×2 (Automation-100%, Automation-80%) ANOVA. The degrees of freedom for all F tests involving repeated measures factors were corrected for violations of the sphericity assumption by using the Greenhouse-Geisser procedure, and the alpha level was set at .05. All tests of simple effects were adjusted using the conservative Bonferroni correction.

### Overall Performance

Table 1 gives the mean values of decision accuracy (% correct) and decision time (s) for each DBH genotype group and condition. The main effect of genotype group was not significant for either decision accuracy, *F*(1, 98) = 4.1, or decision time, *F*(1,98) = 1.93. The main effect of condition was significant for both decision accuracy, *F*(2,196) = 99.76, *p*<.0001, ε = 0.80, η^2^_p_ = 0.50, and decision time, *F*(2,196) = 108.8, *p*<.0001, ε = 0.78, η^2^_p_ = 0.53. The group×condition interaction was not significant for either measure, *F*(2,196) = 0.92, and *F*(2,196) = 2.23, respectively. Overall decision accuracy was higher and decision time was lower in the two automation conditions than in the manual condition, for both genotype groups.

**Table 1:** Mean percentage of correct decisions and mean decision times in seconds (standard deviations in parentheses) in the manual and automation conditions.

Decision Accuracy			
	Manual	Automation-100%	Automation-80%
Low DBH Enzyme Activity	83.9 (7.35)	94.0 (4.61)	89.9 (3.60)
High DBH Enzyme Activity	82.5 (8.19)	93.6 (4.46)	87.6 (2.93)

Decision Time			
Low DBH Enzyme Activity	6.82 (0.76)	6.11 (0.67)	6.1 (0.69)
High DBH Enzyme Activity	7.02 (0.77)	6.15 (0.64)	6.3 (0.73)

### Performance with Imperfect Automation

**Figure 3:**
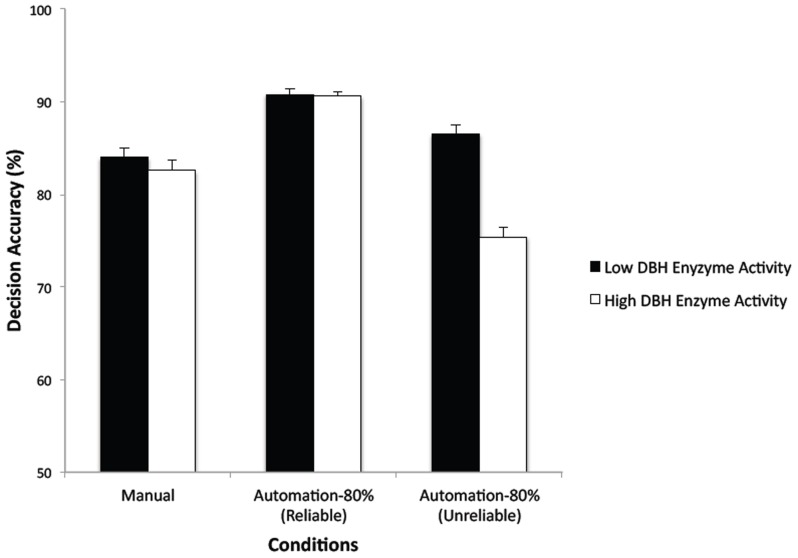
Mean decision accuracy (%) in the Manual condition and on reliable and unreliable trials in the Automation-80% condition. (Bars show standard errors).

**Figure 4:**
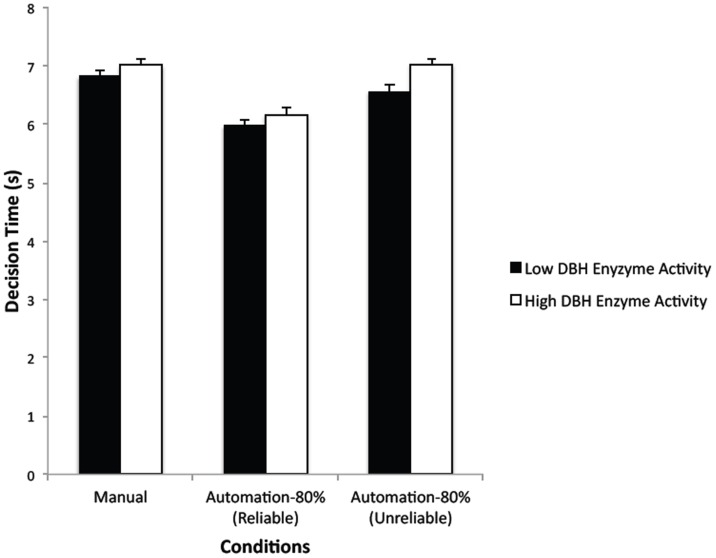
Mean decision time (s) in the Manual condition and on reliable and unreliable trials in the Automation-80% condition. (Bars show standard errors).

The main effect of automation condition (reliable, unreliable), was significant for both decision accuracy, *F*(1,98) = 158.78, *p*<.0001, η^2^_p_ = 0.62, and decision time, *F*(1,98) = 113.19, *p*<.0001, η^2^_p_ = 0.54. Figures 3 and 4 show the mean decision accuracy and time values for the reliable and unreliable automation trials for both genotype groups. Decision accuracy was lower and decision time was higher in the unreliable compared to reliable trials. The main effect of genotype group was significant for both decision accuracy, *F*(1,98) = 44.19, *p*<.0001, η^2^_p_ = 0.31, and for decision time, *F*(1,98) = 62.7, *p* = .01, η^2^_p_ = 0.6. These effects were modulated by a significant group×automation condition interaction, *F*(1,98) = 49.*2*, *p*<.0001, η^2^_p_ = 0.34, for decision accuracy, and *F*(1,98) = 4.55, *p* = .036, η^2^_p_ = 0.04, for decision time.

Figure 3 shows the mean decision accuracies for the two DBH enzyme groups when the task was performed manually and when assisted by 80%-reliable automation. (Note that in Figure 3 “Automation-80% (Reliable)” refers to the 80% of trials on which the automation was correct; “Automation-80% (Unreliable)” refers to the 20% of trials in which the automation gave wrong advice.) As Figure 3 shows, the two groups had equivalent decision accuracy on reliable automation trials, *F*(1,98) = 0.158, *p* = 0.69, but the low DBH enzyme activity group had significantly higher accuracy than the high enzyme activity group on unreliable trials *F*(1,98) = 13.28, *p*<0.0001, η^2^_p_ = 0.12. The high DBH enzyme activity group showed the typical automation bias effect–a reduction in decision accuracy when automation was imperfect–whereas the low enzyme activity group showed a reduced effect. There was a similar pattern of results for decision time. Whereas the two DBH enzyme groups were not significantly different in decision time on the 80% of reliable trials, *F*(1,98) = 0.08, *p* = 0.78, the low enzyme activity group was faster than the high enzyme activity group on the 20% of unreliable trials, *F*(1,98) = 3.92, *p* = 0.05, η^2^_p_ = 0.04 (Figure 4). Given that the low enzyme activity group was also more accurate than the high enzyme group on unreliable automation trials, their lower decision time does not indicate a speed-accuracy tradeoff, but overall more efficient decision making.

**Figure 5:**
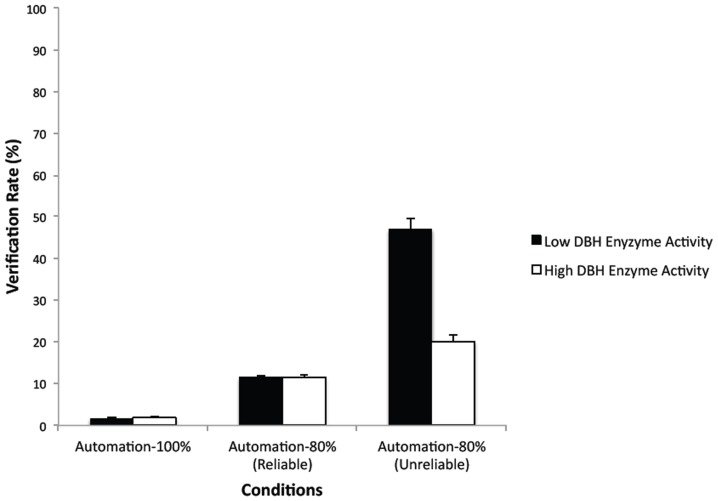
Mean verification rates (%) in the Automation-100% condition and on reliable and unreliable trials in the Automation-80% condition. (Bars show standard errors).

For the verification rate measure, the main effects of genotype group, *F*(1,98) = 79.48, *p*<.0001, η^2^_p_ = 0.45, automation condition, *F*(2,196) = 380.92, *p*<.0001, ε = 0.55, η^2^_p_ = 0.84, and their interaction, *F*(2,196) = 86.83, *p*<.0001, ε = 0.55, η^2^_p_ = .49, were all significant. As Figure 5 shows, both groups had near zero verification rates in the Automation-100% condition, *F*(1,98) = 0.96, *p* = 0.33, and comparable (low) verification rates on reliable trials in the Automation-80% condition, *F*(1,98) = 0.05, *p* = 0.83. However, the low enzyme activity group had a significantly higher (more than twice the) verification rate on unreliable trials than the high enzyme activity group, *F*(1,98) = 90.67, *p*<0.0001, η^2^_p_ = 0.48.

The results for subjective trust were similar to those for verification rate. The effects on trust of genotype, *F*(1,98) = 6.56, *p* = .012, η^2^_p_ = 0.06, automation condition, *F*(1,98) = 111.09, p<.0001, η^2^_p_ = 0.53, and their interaction, F(1,98) = 9.8, *p*<.0001, η^2^_p_ = 0.09, were each significant. Both genotype groups reported similar levels of (high) trust in the automation when it was perfectly reliable, *F*(1,98) = 0.09, *p* = 0.76. In the 80% automation reliable condition, however, the low DBH enzyme activity reported significantly lower levels of trust than the high enzyme activity group, *F*(1,98) = 11.22, *p*<0.05, η^2^_p_ = 0.10.

Finally, the correlation between verification rate and trust was significant, −.64 (*p*<.001). This finding is consistent with the ANOVA results and points to a relationship between objective and subjective measures of trust in automation.

## Discussion

Accuracy on a complex decision-making task involving simulated command and control was reduced when a computerized decision aid provided advice that was only 80% reliable. On the 20% of trials when the automation gave incorrect advice, many (but not all) individuals erroneously went along with the computer decision. This finding is consistent with previous findings indicating that people exhibit automation bias when assisted by imperfect decision aids [4], [36]. We predicted that variants of the DBH gene would be associated with individual differences in the degree to which participants exhibited this bias. Specifically, individuals with gene variants associated with low DBH enzyme activity (high dopamine compared to norepinephrine levels) would show superior decision-making performance compared to those with high DBH enzyme activity under imperfect decision aiding. This hypothesis was supported. While there were no differences in overall decision making accuracy or decision time between the low and high DBH enzyme activity groups when the decision-making task was carried out manually or with perfectly (100%) reliable automation, the low DBH enzyme activity group was more accurate and speedier in making engagement decisions in the Automation-80% condition on those trials when incorrect advice was given. Thus, whereas the high DBH enzyme activity (lower DA level) group showed the typical automation bias effect [4]–a reduction in decision accuracy from 90.6% on reliable trials to 75.4% on unreliable trials, the low DBH enzyme activity (higher DA level) group showed a significantly reduced automation bias–from 90.8% to 86.5%.

These results indicate that a gene that regulates relative dopamine availability in prefrontal cortex, namely the DBH gene, plays a role in inter-individual variation in time-stressed decision-making performance under imperfect automated aiding. Specifically, individuals with variants of the DBH gene with low levels of DBH enzyme activity, which is associated with higher dopamine to norepinephrine levels in cortex [27], [28], exhibit superior decision making in an automated command and control task when incorrect advice is given. Thus, the DBH gene influences the degree to which decision-making performance is adversely affected by biased use of computerized decision aids.

Supporting evidence for this view was provided by the results on information verification rates. The low DBH enzyme activity group, who showed less susceptibility to automation bias, verified automation recommendations on unreliable trials at more than twice the rate of the high DBH enzyme activity group. Moreover, they also reported lower subjective trust in the automation on unreliable automation trials. These findings are consistent with the conclusions of Bahner and colleagues [9] that objective data on verification behavior are needed to determine whether automation biases decision making in complex, dynamic tasks such as command and control and process control. The results for the subjective ratings of trust provided further corroborative evidence: the low DBH enzyme activity reported lower trust in the decision aid on the unreliable trials.

Modulation of task performance by normal variation in the DBH gene may reflect the role of executive functioning in successful decision-making. Executive functioning is claimed to be composed of inhibition, set shifting, and updating in working memory [14]. Of these three, updating in working memory was found in a large twin study to be the most heritable and have the strongest correlation with general intelligence [14]. It has long been established that the binding of dopamine D1 receptors is strongly related to working memory performance in monkeys [25] and humans [24], [40]. Regarding updating in working memory, there is neuroimaging evidence of increased release of dopamine from the striatum related to training aimed at working memory updating [41]. Working memory capacity also appears to be influenced by the striatal dopaminergic system. Working memory capacity has been found to vary with a DRD2 haplotype [42] previously found to modulate both working memory performance and neural activity in striatum and prefrontal cortex during an N-back task [43]. One possible interpretation of our results, therefore, is that the (highly heritable) ability to rapidly update information in working memory–which is associated with higher dopamine levels and variation in the DBH gene [28]–[30]–may influence the time or resources available needed to consider automation recommendations and confirm them in complex decision-making tasks.

A unique contribution of this study is the identification of genetic sources of individual differences in decision making in complex tasks with imperfect automation, and more specifically with automation bias. The present study had a fairly small sample size of 100 individuals, mainly because we selected specific genotype combinations from a larger sample. Replication of the present results, preferably in bigger samples, is necessary before firm conclusions can be reached on the possibility of using genetic findings for selection of individuals who exhibit little or no automation bias, or for training of those who exhibit this tendency to a high degree. Previous studies have found that automation bias occurs in both novices and in expert populations such as pilots [11], and while individual differences have been noted [10], their basis has not been identified. Given that the DBH gene has been linked to executive function and working memory [29], [30], the present results suggest that inter-individual variation in these cognitive functions are major contributing factors.

The results of the present study cannot distinguish between a direct association between the DBH gene and automation bias or an effect that is mediated by individual differences in working memory capacity or executive function. Given our previous findings linking DBH and working memory [29], [30], [44], we favor the mediation interpretation. Furthermore, we have shown that individual differences in working memory capacity are predictive of effective use of automation in a simulated air defense task [45]. Unfortunately, we did not have working memory or executive function test scores on the 100 adults tested in the present study. Whether working memory is a critical mediating factor in individual differences in appropriate use of automation in complex decision-making tasks is an important issue for future research.

The consequences of humans “blindly” accepting incorrect computer advice can at best be undesirable and possibly correctable through training (but see [12]). However, in some instances the outcomes could be severe and in the extreme could lead to loss of life [8]. Given that perfectly reliable automated decision aids cannot be assured [7], there is a need to identify ways to reduce automation bias. Our findings suggest that the DBH gene, which regulates the differential cortical availability of DA and NE, is associated with superior decision making when individuals are assisted with imperfect automated aids. Other genes that influence prefrontal DA levels, such as COMT, have been linked to working memory and executive function, although recent meta-analyses found the associations to be relatively weak and inconsistent [46], [47]. Recently, variants of the COMT and DRD4 genes were reported to predict successful financial decision making by Wall Street traders [48]. Of course, the imperfections of computer-assisted trading were also to blame for the financial crises in the stock market in 2010 [49], suggesting that the effects may have been less severe if traders had been less susceptible to automation bias. Our results have implications for the development of selection and training procedures aimed at forming teams of human operators who can make speedy and accurate decisions that are less biased by imperfect computerized decision aids.
